# Combined Targeted and Non-targeted PCR Based Methods Reveal High Levels of Compliance in Probiotic Products Sold as Dietary Supplements in United States and Canada

**DOI:** 10.3389/fmicb.2020.01095

**Published:** 2020-06-09

**Authors:** Hanan R. Shehata, Steven G. Newmaster

**Affiliations:** ^1^NHP Research Alliance, College of Biological Sciences, University of Guelph, Guelph, ON, Canada; ^2^Department of Microbiology, Faculty of Pharmacy, Mansoura University, Mansoura, Egypt

**Keywords:** probiotic authentication, high-throughput sequencing, viable count, species-specific PCR, strain-specific PCR

## Abstract

Probiotics are defined as “live microorganisms that, when administered in adequate amounts, confer a health benefit on the host.” The diverse health benefits have contributed to rapid increase in probiotic consumption and in the value of probiotic market, valued at USD 46 billion in 2019. For probiotics to be effective, the correct species/strains should be delivered viable in an adequate dose. The most commonly used methods for species/strain identification are DNA based methods including targeted and non-targeted methods (e.g., high-throughput sequencing, HTS). Using different DNA based methods, previous studies reported several cases of non-compliance in probiotic products. The objectives of this study are to evaluate levels of compliance in probiotic products (presence of all declared species/strains, absence of any contaminants or undeclared species, and meeting the declared minimum viable cell count) and to compare the performance of targeted and non-targeted methods in probiotic authentication. To the best of our knowledge, this is the largest study of its kind, testing 182 probiotic products, containing a total of 520 strains, collected from United States and Canada. Using species-specific assays, 11 species could not be detected in ten products. Missing species were *Lactobacillus casei* in seven products, *Bifidobacterium longum* and *Bifidobacterium bifidum* in one product, *B. longum* in one product while *B. longum* subsp. *longum* was mislabeled as *B. longum* subsp. *infantis* in another. Additionally, undeclared *Bifidobacterium animalis* subsp. *lactis* was detected in one product. Viable count was determined for 72 samples and was found to be lower than declared in five samples, including one product showing no viable cells. Overall, non-compliance was observed in 15 out of 182 products (8%). Additionally, undeclared species at relative abundance of ∼1–2% were found in 14 products using HTS, however, their presence could not be confirmed using species-specific assays. The results show that targeted PCR based methods enable species and strain level identification. The results also highlight the need to continue to develop strain-specific assays appropriate for use with multi-strain products. True strain-specific assays will enable strain authentication in both single-strain products and multi-strain products to ensure probiotic products meet the label claims and ensure probiotic efficacy.

## Introduction

Probiotics are defined as “live microorganisms that, when administered in adequate amounts, confer a health benefit on the host” (Hill et al., 2014). Among potential health benefits of probiotics are their role in treating infectious diarrhea, antibiotic associated diarrhea, and irritable bowel syndrome (Ng et al., 2008; Moayyedi et al., 2010; Sánchez et al., 2017; Floch, 2018), their immunomodulatory and anti-allergic effects (Borchers et al., 2009; Bubnov et al., 2015), cholesterol lowering effect (Nguyen et al., 2007), and cancer preventing effect (Rajoka et al., 2017; Sharma et al., 2018). These diverse human health benefits have attracted microbiologists and clinicians (Huys et al., 2006), and have contributed to the rapid growth in probiotic consumption and in the value of probiotic market. The value of global probiotic market was USD 46.20 billion in 2019 and is predicted to increase to USD 75.90 billion by end of 2026 (Zion-Market-Research, 2020).

In order for probiotics to exert their health benefits, the correct species/strains should be delivered viable, and in an adequate dose. Thus, it is important when manufacturing and packaging probiotic products to ensure the presence of the correct species/strains at the appropriate viable count throughout their shelf life (Tripathi and Giri, 2014; Kolaćek et al., 2017; Sánchez et al., 2017). In fact, Joint Food and Agriculture Organization of the United Nations and World Health Organization (FAO/WHO) Working Group recommended that genus, species and strain names and viable count at expiration to be declared on probiotic labels (FAO/WHO, 2002). Health Canada set the guidelines for probiotic labeling to include genus, species, and strain names and cell count at the end of shelf life on product labels when claiming health benefits (Health-Canada, 2009a, b, 2015).

To ensure correct species/strain identification and viable count determination, several methods were developed and have been used for the purpose of probiotic authentication. For species/strain identification, the most commonly used methods are culture-based methods followed by phenotypic and genotypic characterization, and DNA based methods. DNA based methods can be targeted (active) methods (e.g., species-specific and strain-specific PCR) or non-targeted (passive) methods (e.g., high-throughput sequencing, HTS) (Fasoli et al., 2003; Theunissen et al., 2005; Marcobal et al., 2008; Drago et al., 2010; Simmons et al., 2015; Morovic et al., 2016; Patro et al., 2016; Chen et al., 2017; Shehata et al., 2019). For viable count determination, plate count methods are the most commonly used methods for bacterial enumeration (Davis, 2014).

Previous studies conducted in United States, Europe, and China investigated and reported variable rates of non-compliance in probiotic products (Kolaćek et al., 2017). The objectives of this study are to: (1) evaluate levels of compliance in probiotic products in terms of presence of all declared species/strains, absence of any contaminants or undeclared species, and meeting the declared minimum viable cell count and (2) evaluate and compare the performance of targeted and non-targeted methods for probiotic authentication. To the best of our knowledge, this is the largest study of its kind to evaluate levels of compliance in probiotic products, testing a total of 520 strains in 182 probiotic products collected from Canada and United States. This study employed a combination of targeted and non-targeted methods for probiotic authentication.

## Materials and Methods

### Sample Collection and Processing

A total of 182 probiotic products, containing a total of 520 strains in 470 species, were collected from various probiotic manufacturers in USA and Canada (Supplementary Table S1). Among them, 45 products were finished products in their final delivery form and 137 products were powder materials ready for formulation into their final delivery form. Samples were stored either frozen or at room temperature, following the recommended storage conditions. DNA was extracted from all probiotic samples using NucleoSpin Food kit (740945.50, Macherey Nagel, Germany), according to manufacturer’s instructions. DNA was quantified using Qubit 3.0 Fluorometer and was stored in a − 20°C freezer until use.

### Targeted PCR for Declared Species/Strain Verification

The species-specific primers developed by Morovic et al. (2016) were used to verify the presence of all declared species in all test samples. For products containing *Lactobacillus acidophilus* NCFM and La-14, *B. lactis* Bl-04, Bi-07 and HN019, *Lactobacillus paracasei* Lpc-37, or *Lactobacillus rhamnosus* HN001, strain-specific assays were used to confirm the presence of the declared strains (Morovic et al., 2016). Species-specific and strain-specific PCR were conducted following the methods of Morovic et al. (2016). Universal 16S primers (799f and 1492r) were used for one product containing a single strain of *Bacillus coagulans* (Frank et al., 2008; Ghyselinck et al., 2013). Successful amplification was verified by examining all PCR products on bufferless, pre-cast 2% E-gel stained with Ethidium Bromide (G700802, Invitrogen). PCR products were submitted for sequencing at the Advanced Analysis Centre, Genomics facility at the University of Guelph (Guelph, ON, Canada) and sequencing results were analyzed using CodonCode Aligner 7.1.1.

### Non-targeted HTS for Declared Species Verification and Undeclared Species Detection

The V3 and V4 region of 16S rRNA gene was targeted using the following primers:

F: TCGTCGGCAGCGTCAGATGTGTATAAGAGACAGCCT ACGGGNGGCWGCAG and R: GTCTCGTGGGCTCGGA GATGTGTATAAGAGACAGGACTACHVGGGTATCTAATCC (Klindworth et al., 2013; Illumina, 2013). Each reaction mixture (25 μl total volume) consisted of 12.5 μl of 2× KAPA HiFi Hotstart Ready Mix (07958935001, Roche), 2.5 μl of DNA (5ng/μl), 5 μl from each of the primers (1 μM working solutions). Thermal cycling conditions were 95°C for 3 min; 25 cycles of: 95°C for 30 s, 55°C for 30 s, and 72°C for 30 s; followed by 72°C for 5 min and a hold at 4°C. PCR amplicons were cleaned using AMPure XP beads (A63881, Beckman Coulter) as described in Illumina protocol (Illumina, 2013). PCR products were eluted in 10 mM Tris pH 8.5 and used in index PCR. Each index PCR reaction (50 μl total volume) consisted of 25 μl of 2× KAPA HiFi Hotstart Ready Mix, 10 μl of PCR grade water, 5 μl each from Nextera XT index primer 1 and index primer 2, and 5 μl of cleaned PCR product. The thermal cycling conditions were 95°C for 3 min, 8 cycles of 95°C for 30 s, 55°C for 30 s, and 72°C for 30 s, followed by 72°C for 5 min and a hold at 4°C. PCR products were purified using AMPure XP beads and eluted in 25 μl of 10 mM Tris pH 8.5. Samples were normalized, pooled and sequenced using a MiSeq v3 600 cycle kit, on a 2 × 300 bp run at the Advanced Analysis Centre, Genomics facility at the University of Guelph (Guelph, ON, Canada).

Sequencing results were analyzed using CLC Genomics Workbench (QIAGEN Bioinformatics). FASTQ files were imported and merged (mismatch cost = 2, minimum score = 8, gap cost = 3 and maximum unaligned end mismatches = 0). Reads were quality trimmed (quality score limit set to 0.05 and maximum number of allowed ambiguities set to 0) and primer sequences were also trimmed. Reads were then trimmed to a fixed length (407 b) and were used for operational taxonomic unit (OTU) assignment using SILVA 16S v132 97% reference OTU database with similarity percentage set to 97% (Yilmaz et al., 2013). Operational taxonomic units with low abundance, represented by less than 0.005 of total reads per sample, were excluded to ease data analysis.

### Estimating Total Viable Count

The total viable count was determined for 72 samples using pour plate technique. From each sample, 1 g of powders or one dose of finished products was suspended in 10 ml of peptone water (70179, Sigma) to achieve 10% (w/v) suspensions. These suspensions were vortexed, and incubated at room temperature for 30 min to allow rehydration. Suspensions were vortexed again and then 10-fold serial dilutions were prepared until a dilution corresponding to 20–200 colony forming units (CFU)/ml, based on declared cell count, was reached. MRS agar (69964, Sigma) supplemented with 0.05% L-cysteine hydrochloride monohydrate (C7880, Sigma) was prepared and cooled to 45°C. In triplicate Petri dishes, 15 ml of MRS agar were mixed with 1 ml of the highest dilution of probiotic suspensions (pour plate technique). Agar was allowed to solidify then plates were inverted and incubated in AnaeroPack Jar (23-246-387, Fisher) with AnaeroPack Anaerobic Gas Generator (23-246-376, Fisher) at 37°C for 72 h. CFUs per plate were recorded and averaged. Total viable count in probiotic products was determined by multiplying plate count by dilution factor.

## Results

### Sample Collection

Among 182 probiotic products, 132 products contained a single strain while 50 products contained multiple strains ranging from 2 to 14 strains per product (Supplementary Table S1). The number of strains in 50 multi-strain products was 388 strains and the total number of strains in all 182 products was 520 strains in 470 species. The products were in different forms (capsules, *n* = 35, chewable tablets, *n* = 4, gummies, *n* = 1, drops, *n* = 2, powder sticks, *n* = 2, finished powder, *n* = 1, powder, *n* = 137) (Supplementary Table S1).

### Targeted PCR for Declared Species/Strain Verification

**FIGURE 1:**
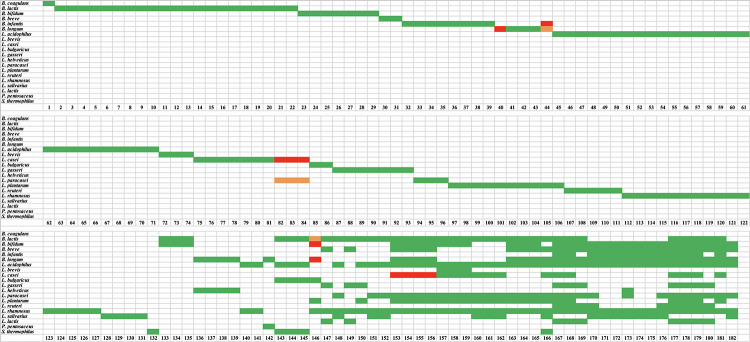
Results of targeted species-specific PCR testing of probiotic samples. Green indicates a species was declared and present. Red indicates a species was declared but absent. Orange indicates that a species was not declared but was present.

**FIGURE 2:**
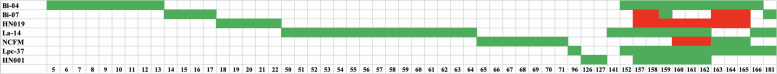
Results of targeted strain-specific PCR testing of probiotic samples. Green indicates a strain was declared and present. Red indicates a strain was declared but could not be detected using these assays.

A total of 519 strains were tested using species-specific and/or strain-specific assays (Morovic et al., 2016) and one strains of *B. coagulans* was tested using universal 16S primers (799f and 1492r) (Frank et al., 2008; Ghyselinck et al., 2013). For the 132 single-strain products, the presence of the target species was confirmed in all products, except five products (sample number 40, 44, 82, 83, and 84) (Figure 1). Product 40 was labeled to contain *Bifidobacterium longum*, however, no amplicon was obtained with *B. longum* species-specific primers. Product 44 was labeled to contain *B. longum* subsp. *infantis*, however, it was found to contain *B. longum* subsp. *longum*. Products 82, 83, and 84 were labeled as *Lactobacillus casei*, however, they were found to contain *L. paracasei*. Strain-specific assays were available for only 43 of the 132 single-strain products. All 43 strains were positive (Figure 2). The results from targeted PCR show that 43 products were positive while 89 were potentially positive since they were identified to species level only.

For the 50 multi-strain products, the presence of target species was confirmed for 332 out of 338 species (Figure 1) and these were count as potentially positive. The six missing species were *L. casei* in four products (samples 153, 154, 155, and 156), and *B. longum* and *Bifidobacterium bifidum* in sample 146. Additionally, the presence of 44 strains was confirmed using strain-specific assays (Figure 2).

### Non-targeted HTS for Declared Species Verification

**FIGURE 3:**
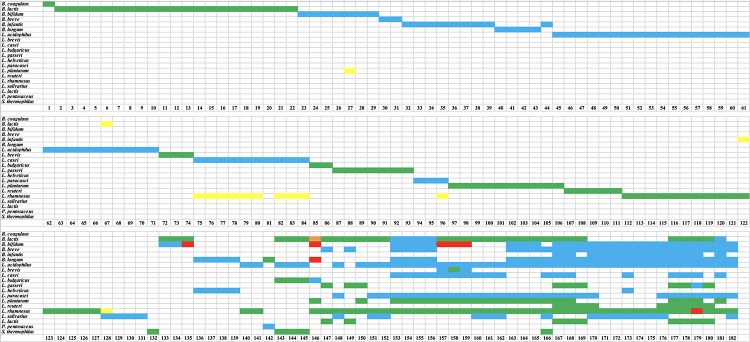
Results of high throughput sequencing (HTS) of probiotic samples. Green indicates that a declared species was present. Blue indicates that a declared species was identified to genus level only. Red indicates a species was declared but absent. Orange indicates that a species was not declared but was present. Yellow indicates that a species was not declared but was present at low relative abundance (∼1–2%).

The use of HTS in this study confirmed the presence of target species/genus in all 132 single-strain products (Figure 3). Among 132 target species/strains, 66 targets were identified to species level while 66 targets were assigned to genus level only (*L. acidophilus, L. casei, L. paracasei*, and *L. salivarius* were identified as *Lactobacillus* sp. while *B. bifidum*, *B. breve*, and *B. longum* were identified as *Bifidobacterium* sp.). The relative abundance of the target species, based on read numbers, was ∼100% in 95 samples, ∼99% in 26 samples, and ∼98% in 11 samples.

For the 50 multi-strain products, HTS confirmed the presence of all declared genera/species, with the exception of seven species in six products (Figure 3). The species that were missing based on HTS were *B. bifidum* in products 135, 157, 158, and 159, *Lactobacillus rhamnosus* in product 179, and *B. longum* and *B. bifidum* in product 146 (Figure 3).

### Non-targeted HTS for the Detection of Undeclared Species

Undeclared species were found in 14 of 132 single-strain products at relative abundance of ∼1–2% (Figure 3). Eleven of these 14 cases were undeclared *L. rhamnosus* in *L. casei, L. paracasei*, or *L. salivarius*. The other three cases were: *Bifidobacterium animalis* in *L. acidophilus* (1% relative abundance), *Bifidobacterium* sp. in *L. rhamnosus* (1% relative abundance), and *Lactobacillus plantarum* in *B. bifidum* (2% relative abundance). For multi-strain products, undeclared *B. animalis* was found in one product (sample 146) at 15% relative abundance.

### Estimating Total Viable Count

**FIGURE 4:**
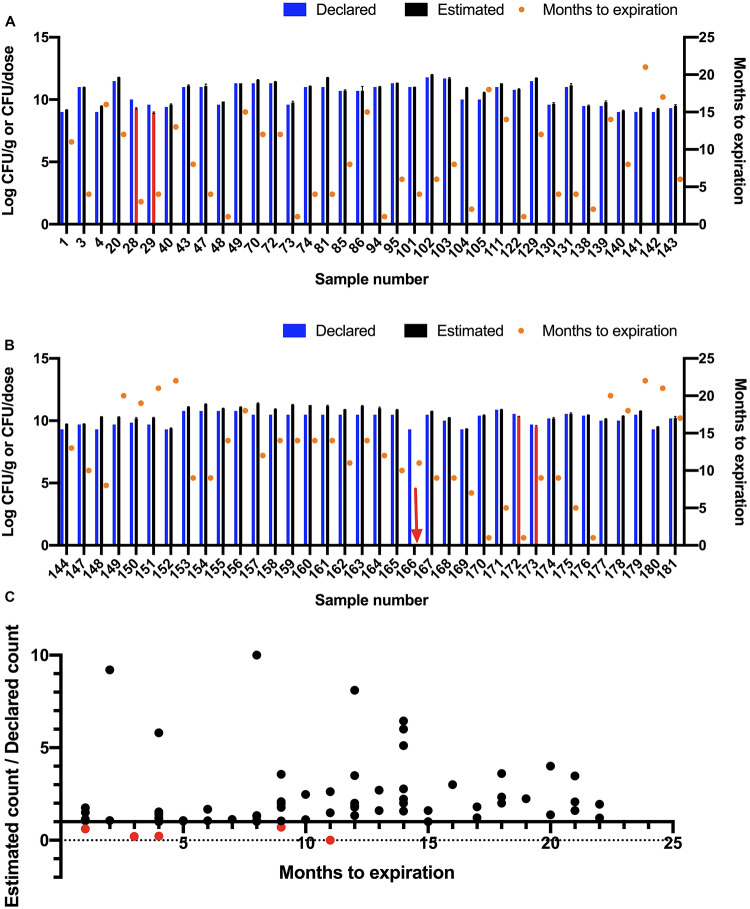
Estimating colony-forming units (CFU) of probiotic samples using a plate culture method. **(A,B)** Declared and estimated viable count where Log_10_ estimated CFU/g or CFU/dose are shown on left *Y*-axis as the mean of three replicates with Standard Error of Mean (SEM). The bars are shown in red for samples that did not meet the declared viable counts. The red arrow indicates a sample that did not show any viable count. The declared viable counts are shown as blue bars. Time to expiration in months is shown on right *Y*-axis (orange circles). Samples are split into two graphs for ease of readability. **(C)** Ratio of estimated viable count to declared viable count compared to months to expiration at time of testing.

Total viable count was estimated for 72 samples using a plate count method (Figure 4). The viable count in 67 samples (93%) met or exceeded the declared viable count. The viable count in four samples (28, 29, 172, and 173) was lower than the declared viable count and no growth was observed for one sample (166). The five samples (7%) that did not meet label viable count claims were re-tested and were found to be below label claims in both trials. All five samples were tested before their expiration dates.

## Discussion

Because health benefits and safety of probiotics are strain specific, it is important to ensure that probiotic products contain the correct strains to achieve their intended health benefits. It is equally important to ensure that probiotic products contain the minimum declared viable count of probiotics throughout shelf life (Tripathi and Giri, 2014; Kolaćek et al., 2017; Sánchez et al., 2017). The most commonly used methods for probiotic authentication are culture-based methods and DNA based methods. In this study, DNA based methods including targeted (species-specific and strain-specific PCR) and non-targeted methods (HTS) as well as a culture based method were used to evaluate compliance in probiotic products sold as dietary supplements in United States and Canada. The results show high levels of compliance in probiotic products. The level of compliance found in this study is higher than those reported from previous studies, which may be attributed to the advancements in sequencing techniques and the improvements in DNA based methods for species and strain identification.

### Targeted PCR for Declared Species/Strain Verification

Using species-specific assays, the presence of target species was confirmed in 127 out of 132 single-strain products. Three products were declared as *L. casei* but were found to be *L. paracasei*. *L. casei* and *L. paracasei* are very closely related species and the high genetic similarity may have lead to the misidentification of *L. paracasei* as *L. casei* in these three products. Another possibility is that strain manufacturers may have used *L. casei* to refer to the larger *L. casei* group which includes both *L. casei* and *L. paracasei*, in addition to *L. rhamnosus* (Huang et al., 2018). Although, *L. paracasei* was previously referred to as *L. casei* subsp. *paracasei* (Morovic et al., 2016), *L. paracasei* is the valid name (The Integrated Taxonomic Information System)^1^.

Another product was labeled as *B. longum*, however, it was negative when tested using *B. longum* species-specific primers. This product also contained cranberry fruit (*Vaccinium macrocarpon*). Cranberry is known to be rich in polyphenols (Flammer et al., 2013), which are known to inhibit PCR (Schrader et al., 2012). We hypothesized that the failure of PCR amplification in this sample may be attributed to PCR inhibition, rather than absence of the target species and that additional testing using HTS will prove or disprove this hypothesis. The fifth product was labeled to contain *B. longum* subsp. *infantis*, however, it was found to contain *B. longum* subsp. *longum*. Similar cases of mislabeling were previously reported where *B. longum* subsp. *longum* was declared as *B. longum* subsp. *infantis* (Lewis et al., 2016; Patro et al., 2016). These two subspecies of *B. longum* are very closely related which makes it difficult to differentiate, leading to possible misidentification (Lewis et al., 2016).

Species-specific assays were positive for 332 out of 338 species in 50 multi-strain products. The six missing species were *L. casei* in four products and *B. longum* and *B. bifidum* in another sample. The four cases where *L. casei* was missing may be similar to the three cases of single strain products where *L. casei* was substituted with *L. paracasei.* Since all of these four multi-strain products were declared to contain *L. paracasei* in addition to *L. casei*, the use of species-specific PCR targeting *L. paracasei* to test this hypothesis was not possible.

The absence of *B. longum* and *B. bifidum* in one sample may be attributed to poor stability. Stability and viability of *Bifidobacterium* species during processing and storage represent a big challenge for industry (Celik and O’Sullivan, 2013). For example, *B. longum* is known to be sensitive to adverse environmental conditions including temperature, humidity and oxygen, which may result in reduced viability and stability during production and storage (Ji et al., 2019).

Overall, 459 out of 470 species in 182 products were potentially positive as they were confirmed to species level only. Eleven species could not be verified in ten out of 182 (5.5%) tested probiotic products.

In single-strain products, strain-specific assays were positive for all 43 tested samples while for multi-strain products, strain-specific assays were positive for 44 out of 61 tested samples. Failure of 17 strain-specific assays can be attributed to the lack of true strain-specific methods that are appropriate for use with multi-strain products especially blends containing closely related strains. Strain-specific assays used here for *B. animalis* subsp. *lactis* and *L. acidophilus* strains rely on SNP identification, limiting their utility with probiotic blends. For example, six products contained three strains of *B. animalis* subsp. *lactis* and three products contained two strains of *B. animalis* subsp. *lactis*, and two strains of *L. acidophilus.*

Additionally, 13 of the products contained two strains of *L. rhamnosus.* Using species-specific primers, the presence of two distinct strains could not be verified. In general, it is much straightforward to test the individual strains prior to blending to avoid cross reactivity from closely related strains present in a blend. Alternatively, developing and validating true strain-specific assays that can detect closely related strains in blends would be ideal to allow and facilitate testing of probiotic strains whether in single-strain or multi-strain products.

### Non-targeted HTS for Declared Species Verification

HTS confirmed the presence of target species/genus in all 132 single-strain products where 66 targets were identified to species level and 66 targets were assigned to genus level only. For multi-strain products, HTS confirmed the presence of all declared species/genera, except for seven species in six products, which were *B. bifidum* in four products, *L. rhamnosus* in one product, and *B. longum* and *B. bifidum* in one product. Poor taxonomic resolution and inaccurate identification are expected in amplicon based HTS due to the short read length (Zaura, 2012). Due to the poor taxonomic resolution, target species were identified to genus level only in many cases, which made it difficult to get information on whether the different species declared on a label existed or not. The relative abundance of the target species in single-strain products ranged from ∼98 to 100%. It is important to note that read abundance from HTS may not accurately reflect the biological relative abundance in the sample due to potential biases during DNA extraction and PCR amplification (Shokralla et al., 2012; Zaura, 2012; Pawluczyk et al., 2015). Among the seven missing species, two species (*B. longum* and *B. bifidum* in one product) were also found to be missing using the targeted assays. The other five species that were missing in HTS (*B. bifidum* in four products and *L. rhamnosus* in one product) were positive using the targeted assays. Perhaps these species existed at very low percentages in the blends that they were not detected by HTS or were detected but excluded during data de-noising, where OTUs represented by less than 0.005 of the reads in a sample were excluded.

### Non-targeted HTS for the Detection of Undeclared Species

HTS revealed the presence of undeclared species in 14 of 132 single-strain products at relative abundance of ∼1–2%. The presence of undeclared species at very low relative abundance may suggest erroneous sequences or contamination. Eleven of these 14 cases were undeclared *L. rhamnosus* in *L. casei, L. paracasei*, or *L. salivarius*. Indeed, 9 out of 10 samples declared as single species of *L. casei* were also found to contain *L. rhamnosus*. This systematic association suggests that the presence of undeclared *L. rhamnosus* may originate from erroneous sequences as a result of PCR or sequencing errors (Pfeiffer et al., 2018) or from erroneous identification since *L. casei, L. paracasei*, and *L. rhamnosus* are closely related species, all belonging to the *L. casei* group (Huang et al., 2018), rather than physical product contamination. To find out whether these undeclared species were true contaminant or not, species-specific primers targeting these undeclared species were used and none of the undeclared species was positive, supporting the hypothesis that these species resulted from erroneous sequences or erroneous identification.

On the other hand, undeclared *B. animalis* was found in one multi-strain product (sample 146) at 15% relative abundance. Indeed, the presence of *B. animalis* in this sample was confirmed using species-specific primers. The high relative abundance of undeclared *B. animalis*, along with the absence of two other declared species in this sample suggests potential species substitution. The two missing species in this sample are *B. longum* and *B. bifidum*, which are known to be sensitive to adverse environmental conditions (Celik and O’Sullivan, 2013; Ji et al., 2019). This may explain species substitution in this sample.

### Processing of HTS Data

As any other sequencing technique, sequencing errors can occur in HTS. In addition to pre-processing of sequencing data (quality filtration, read length threshold, and ambiguous base calls), further filtration is recommended to de-noise the sequencing data (Kunin et al., 2010; Zaura, 2012). Additional de-noising is, however, associated with the risk of losing real but low abundance taxa (Zaura, 2012). Multiple methods are proposed in the literature for de-noising (Laehnemann et al., 2015). The de-noising method used in this study excluded OTUs represented by less than 0.005 of the reads in a sample.

SILVA 16S v132 97% reference OTU database was used to assign OTUs in this study (Yilmaz et al., 2013). The identities of two OTUs from SILVA were found to be different from the identities obtained when OTU sequences were BLAST searched on GenBank. The first OTU was identified as *Lactobacillus pentosus* but was identified as *L. pentosus* or *L. plantarum* on GenBank. The second OTU was identified as *Streptococcus* sp. but was identified as *Lactobacillus delbrueckii* on GenBank. GenBank identities were used in this study.

### Comparing Targeted and Non-targeted Methods for Declared Species Verification

In general, the results from targeted and non-targeted methods agreed where target species/genus were verified using both methods for 127 out of 132 single strain products and for 327 out of 338 species in multi-strain products. Additionally, both methods could not detect two declared species (*B. longum* and *B. bifidum)* in sample 146. In this sample, non-targeted HTS revealed the presence of undeclared *B. animalis* (15% relative abundance). This sample was then tested using *B. animalis* species-specific primers and the presence of this undeclared species was confirmed again using the targeted assay.

In some cases the results of targeted and non-targeted methods did not agree. For example, the targeted assay was negative for *B. longum* in sample number 40. However, HTS confirmed the presence of *Bifidobacterium* sp. (100% relative abundance) in this sample. Given the poor taxonomic resolution of HTS technique used in this study, this sample was considered inconclusive since the species was identified to genus level only. Similarly, *L. casei* could not be detected using targeted species-specific assay in seven samples (82, 83, 84, 153, 154, 155, and 156) while HTS revealed the presence of an OTU identified as *Lactobacillus* sp. in all seven products. In product 44, declared *B. longum* subsp. *infantis* was found to be *B. longum* subsp. *longum* using targeted assay while HTS revealed the presence of an OTU identified as *Bifidobacterium* sp. in this sample. As HTS has poor resolution at species level, it could not differentiate between *L. casei* and *L. paracasei* or between *B. longum* subsp. *infantis* and *B. longum* subsp. *longum* as did the targeted assays. Five species (*B. bifidum* in four products and *L. rhamnosus* in one product) that were not detected by HTS in multi-strain products were positive in targeted assays.

### Previous Studies Investigating Accuracy of Declared Species/Strains in Probiotic Products

Previous studies have used targeted or non-targeted methods to investigate the accuracy of label claims and have reported variable rates of non-compliance. Fasoli et al. (2003) used species-specific PCR and PCR-DGGE to test seven probiotic products from Italy and found that some species were missing while some undeclared species were detected. Theunissen et al. used species-specific PCR and PCR-DGGE to test nine probiotic products from South Africa and reported that only three products (33.3%) contained the declared species (Theunissen et al., 2005). Marcobal et al. (2008) used species-specific PCR to test 14 probiotic products from the USA, which verified the presence of the declared species. In 2013, Toscano et al. used species-specific PCR to test 24 products from Italy and Europe and identified contamination with *Enterococcus faecium* in two products as well as missing species in two products (Toscano et al., 2013). Chen et al. (2017) used Gram staining, biochemical testing, colony morphology and PCR based methods to test eight probiotic products from China and reported that all eight products were missing some species, contained undeclared species or both. Patro et al. (2016) used whole genome sequencing to test ten probiotic products from USA and reported that all products, except one product contaminated with *Enterococcus*, matched label claims.

Morovic et al. (2016) used targeted PCR based methods and high throughput sequencing to test 52 probiotic products and reported that missing species and/or undeclared species were found in 22 samples. Targeted PCR methods used in the current study are similar to the methods used by Morovic et al. (2016). Some findings were also similar from both studies such as misidentified *B. longum* subsp. *longum* and *B. longum* subsp. *infantis* or misidentified *L. paracasei* and *L. casei* in probiotic products. Additionally, both studies found that strain-specific assays that rely on SNP identification do not perform well when closely related strains exist in the same blend.

While all of the studies described above were conducted on samples collected from the market, two studies by Huys et al. (2006) and Aureli et al. (2010) were conducted on samples collected from producers, distributors, and processing plants. The study by Huys et al. was conducted on 121 probiotic cultures and used DNA and protein profiling methods and reported an overall misidentification rate of 28% (Huys et al., 2006). The study by Aureli et al. used PCR based methods to test 41 probiotic products and reported missing species in 19 of the 41 samples. Furthermore, spores of the pathogenic *Bacillus cereus* were found in one product and spores of *B. subtilis* were found in three products (Aureli et al., 2010).

It is obvious that the level of mislabeling identified in the current study is lower than mislabeling rates reported by previous studies. One possible explanations for this is the recent advancement and affordability in sequencing techniques which enabled collection of genome data for probiotic strains, which in turn enabled the development of reliable identification methods that help manufacturers to accurately identify the species and strains used in the different products. For example, the methods developed by Morovic et al. (2016) proved to be very reliable and accurate for species level identification of probiotics. The difference in mislabeling rate can also be attributed to the difference in the region where test samples were collected. Samples tested in this study were collected from USA and Canada whereas most previous studies were conducted on samples collected from United States, Europe, and China.

### Estimating Total Viable Count

The estimated viable counts in five of the 72 tested products were below the label claims, including one sample (166) that did not show any growth (Figures 4A,B). This sample was tested 11 months before date of expiration. According to the label of this sample, the recommended storage condition to maintain potency is refrigeration. Loss of viable count may have been caused by inappropriate storage conditions. Sample 172 was tested 1 month before expiration and was found to contain 61% of the declared viable count. Samples 28 and 29 were tested 3 and 4 months before expiration dates, respectively and were found to contain 20 and 22% of declared viable counts, respectively. Sample 173 was tested 9 months before expiration and was found to contain 70% of the declared viable count.

The correlation between estimated viable count relative to declared viable count and time to expiration at time of testing was investigated (Figure 4C). No significant correlation was found (*R* square was 0.02547 and *P*-value was 0.1806). A similar finding was reported by Morovic et al. (2016).

Probiotic products should maintain the declared viable count until the expiration date to ensure their probiotic efficacy. Maintaining probiotic viability throughout its shelf life is a major challenge that is facing probiotic manufacturers (Morovic et al., 2016). A number of studies investigated the viable cell count as compared to declared viable cell count on probiotic product labels. Among these studies were four studies conducted on samples collected from the United States market. Drago et al. (2010) tested 13 commercial probiotic products and found that six samples (46%) contained lower viable counts than declared. Goldstein et al. (2014) evaluated the viable count in five probiotic products and reported that the estimated viable counts matched the declared viable cell count in all of the products. Morovic et al. (2016) reported that 17 out of 52 (33%) probiotic products did not meet the declared viable cell count. Patro et al. (2016) estimated the viable cell count in ten probiotic products and reported that only one product (10%) did not meet the declared cell count. Additionally, three studies were conducted on samples collected from the Italian and/or European market. Fasoli et al. (2003) tested seven probiotic products and found that the estimated count was lower that the declared count in all products. Aureli et al. (2010) tested 41 probiotic products and found that only 25 products (61%) met label claim of cell count. Toscano et al. (2013) evaluated the bacterial count in 24 products and found that 10 out of 24 products (42%) did not meet label claim of viable cell count. Another study by Chen et al. (2017) studied eight probiotic products collected in China and found that no viable cells were detected in two of the products (25%). These studies reported variable rates of non-compliance in cell count claims ranging from 0 to 100%. All of these studies used plate count methods to determine viable cell counts, however, they were conducted on samples collected from different countries. It is important to note that these studies, except two studies, were conducted on a limited number of samples (<25 samples).

### Issues With Probiotic Nomenclature

Besides the issues identified above, a number of issues with species nomenclature was noticed in the tested samples. For example, some samples were labeled as containing *B. infantis*. *B. infantis* is an invalid name. The correct name is *B. longum* subsp. *infantis*. Similarly, the names *B. lactis* and *L. bulgaricus* are invalid and should be labeled as *B. animalis* subsp. *lactis* and *L. delbrueckii* subsp. *bulgaricus*, respectively (The Integrated Taxonomic Information System, see footnote 1).

## Conclusion

The overall mislabeling rate determined in this study was lower than that reported from previous studies, which could be attributed to recently developed methods for probiotic authentication such as the methods developed by Morovic et al. which enabled accurate identification of probiotic species. High-throughput sequencing results suggest that HTS protocol used here is not an appropriate tool for probiotic strain identification given its poor taxonomic resolution. High-throughput sequencing, however, enable the detection of contaminant or undeclared species. On the other hand, targeted PCR based methods offer higher taxonomic resolution enabling strain level identification. Lack of true strain-specific methods that are appropriate for use with multi-strain products containing closely related strains shows the need to continue to develop strain-specific assays appropriate for use with multi-strain products. The recent advancement and affordability in sequencing techniques for whole genome sequencing make it feasible to develop reliable strain-specific identification methods. Such methods will enable probiotic strain authentication before blending or after blending to confirm compliance and hence efficacy in probiotic products.

## Data Availability Statement

The data was deposited in the Sequence Read Archive in NCBI under accession number PRJNA611799.

## Author Contributions

HS designed the study, carried out the experiments, analyzed the data, and wrote the manuscript. SN helped to design the study, facilitated sample acquisition, and edited the manuscript. All authors read and approved the manuscript.

## Conflict of Interest

The authors declare that the research was conducted in the absence of any commercial or financial relationships that could be construed as a potential conflict of interest.

## Footnotes

1 https://www.itis.gov

## Supplementary Material

TABLE S1 Sample forms and declared species or strains.
